# Analysis of antibiotic resistance gene cassettes in a newly identified *Salmonella enterica* serovar Gallinarum strain in Korea

**DOI:** 10.1186/s13100-023-00292-8

**Published:** 2023-04-24

**Authors:** Thanh Quang Tran, Minyoung Park, Jong Eun Lee, Soo Hyun Kim, Jae-Ho Jeong, Hyon E. Choy

**Affiliations:** 1 Departments of Microbiology, Basic Medical Research Building, Chonnam National University Medical College, 322 Seoyangro, Hwansun, Jeonnam, 519-763 South Korea; 2 DNA Link, Inc, Seodaemun-Gu Bugahyeon-Ro 150, Industry Coop Bldg. 2Nd Fl, Seoul, 120-140 South Korea; 3 Department of Microbiology, Chonnam National University Medical School, Hwasun, 58128 Jeollanam-Do Republic of Korea

**Keywords:** *Salmonella enterica* serovar Gallinarum, Antibiotic resistance, Transposable element, Transcription

## Abstract

Antimicrobial resistant pathogens are a global health threat driven by the indiscriminate use of antimicrobials. Antimicrobial resistance can be acquired by resistance genes encoded by mobile genetic elements. In this study, we identified a strain of *Salmonella enterica* serovar Gallinarum (SG4021) from an infected chicken in Korea and characterized the presence of resistance genes in its plasmid by whole genome sequencing. The sequence was then compared with that of a plasmid (P2) from strain SG_07Q015, the only other strain of *S.* Gallinarum isolated in Korea for which a genome sequence is available. The results revealed that both strains harbored nearly identical DNA carrying antibiotic resistance gene cassettes inserted into integron In2 of the transposable element Tn*21*, namely an *aadA1* resistance gene conferring resistance to aminoglycosides and a *sul1* resistance gene conferring resistance to sulfonamide. Interestingly, despite the presence of *sul1* in SG4021, an antibiotic sensitivity test revealed that it was sensitive to sulfonamides. Further analysis revealed that this disparity was due to the insertion of a ~ 5 kb ISCR16 sequence downstream of the promoter driving *sul1* expression in SG4021. Using various mutants, we showed that the insertion of ISCR16 blocked the expression of the *sul1* gene from the upstream promoter. Therefore, the functionality of antimicrobial resistance genes determines phenotypic antimicrobial resistance.

**Supplementary Information:**

The online version contains supplementary material available at 10.1186/s13100-023-00292-8.

## Introduction

Antimicrobial resistance in bacteria is driven by multiple genes that are often encoded by mobile genetic elements known as gene cassettes. Gene cassettes can transpose into or out of a specific receptor site (*attI site*), which is mediated by a site-specific recombinase (Int). In many cases, dissemination of antibiotic resistance is aided by the integration of resistance genes into integrons, a phenomenon first observed in the Tn*21* transposon [1]. Tn*21* carries multiple resistance gene cassettes including genes encoding aminoglycoside-modifying enzymes and a mercury resistance (*mer*) operon conferring resistance to environmental mercury. Tn*21* also contains an integron, In2, which encodes an *aadA1* gene cassette conferring resistance to streptomycin/spectinomycin, and a *sul1* gene encoding a modified dihydropteroate synthase that is insensitive to sulfonamides [2], in addition to other insertion sequences.

In Korea, the widespread application of antimicrobial drugs for the treatment of systemic bacterial infections in poultry farms has led to the increased resistance of *Salmonella enterica* serovar Gallinarum (*S.* Gallinarum) to antimicrobial agents [3, 4]. *S.* Gallinarum is a fowl-adapted pathogen that causes typhoid fever in chickens [5]. Recently, *S.* Gallinarum isolates collected from poultry farms that experienced fowl typhoid outbreaks from 2013 to 2018 in South Korea were analyzed [6]. All isolates exhibited a multi-drug resistant phenotype as evidenced by their resistance to at least three of the 18 antimicrobial agents tested. The isolates were particularly resistant to nalidixic acid, ciprofloxacin, and gentamicin, reflecting the high use of antimicrobials such as β-lactams, aminoglycosides, and fluoroquinolones, on poultry farms [6]. This is because most common antimicrobials active against Gram-negatives are chlortetracycline, oxytetracycline, streptomycin, and gentamicin [7, 8]. Therefore, the increased resistance of *S.* Gallinarum isolates can be attributed to the persistent use of these antimicrobials. By contrast, none of the isolates were resistant to eight other antimicrobials including trimethoprim/sulfamethoxazole (sulfonamide), suggesting that the use of these antimicrobials was restricted on poultry farms. In this study, we identified a strain of *S.* Gallinarum (SG4021) isolated from an infected chicken in Korea and compared its whole genome sequence with that of another *S.* Gallinarum strain, SG_07Q015, also isolated in Korea [9]. Both strains harbored virtually identical DNA carrying antibiotic resistance gene cassettes inserted into intergron In2 of Tn21. Interestingly, SG_07Q015 was resistant to sulfonamide but SG4021 was not, although both carried *sul1* resistant genes in In2. Further study revealed that *sul1* in SG4021 was not expressed presumably because of the discontinuation of sulfonamide use on Korean poultry farms.

## Materials and methods

### Bacterial strains

*S*. Gallinarum SG4021 and its derivative strains used in this study are listed in Table 1 and further described in Figure S1. SG4021 genomic DNA was sequenced by Inc. DNA Link (Seoul, Korea). The assembled genome of SG4021 contained two contigs, consisting of one circular genome (4,624,182 bp) and one plasmid (112,953 bp). *S.* Gallinarum SG07Q015 was kindly provided by Dr. H.S. Seo at the Research Division for Radiation Science, Korea Atomic Energy Research Institute, Jeongeup, South Korea.

**Table 1:** Salmonella enterica serovar Gallinarum strains used in this study

Strains	Description	References or sources
SG4021	Wild-type isolate	This work
TH1032	SG4021 pTH1000_Δ_(*Pc-P2)*::*cat*	This work
TH1033	SG4021 pTH1000_Δ_(*aadA1-insF)*::*cat*	This work
SG07Q015	Wild-type isolate	[9]

### Bacterial strain construction

All bacterial mutants were constructed using the λ red recombination method developed by Datsenko and Wanner [10]. Briefly, constructs carrying the *cat* gene flanked by 50 nt target regions for homologous recombination in either the promoter region or desired antibiotic cassettes were generated by PCR amplification using plasmid pKD3 as a template (Table S1). PCR products were purified and treated with DpnI (NEB). Wild-type bacteria carrying a λ red helper plasmid (pKD46) were grown in LB broth (MB Cell) with ampicillin (100 µg/mL) at 30 °C to an optical density at 600_ nm_ of ~ 0.3. L-arabinose was then added to a final concentration of 0.2%, and cultures were allowed to grow for an additional 1 h. Cultures were subsequently washed three times with ice-cold deionized water to generate electrocompetent cells. PCR products were transformed by electroporation, before transformed bacteria were spread on LB-agar supplemented with chloramphenicol (17 µg/mL) and incubated at 37 °C overnight. Mutant strains were confirmed by colony PCR using a pair of primers that bound outside regions of recombination (Table S2).

### Antibiotic sensitivity

To determine sensitivity to antibiotics, overnight bacterial cultures grown at 37 °C in Mueller–Hinton broth (BD Difco) were diluted in warm Mueller–Hinton broth with 0.7% agar to a McFaarland score of 0.5–0.7. The mixture was poured into Mueller–Hinton supplemented with 1.5% agar and left on bench for 10 min. A 25 µg sulfamethoxazole/trimethoprim 1:19 disk (SXT25, Oxoid), 10 µg streptomycin disk (S10, Oxoid), or 10 µg gentamycin disk (CN10, Oxoid) was subsequently applied to the plate. All plates were incubated at 37 °C for 16 h. Inhibition zone diameters were measured by vernier caliper. The strains were identified as susceptible, intermediate susceptible, or resistant based on criteria recommended by the Clinical and Laboratory Standards Institute (CLSI).

### Quantitative Real-time PCR

The 20 µL qPCR mixes consisted of template cDNA (1 µL), a forward primer (1 µL), a reverse primer (1 µL), and 2X qPCR PreMix (10 µL) (Enzynomics, TOPreal™ qPCR 2X PreMix, SYBR Green with lox ROX). Analysis was carried out with a Rotor-GenQ real-time PCR system (Qiagen, Rotor-GenQ series software version 2.2.3). Forty PCR cycles were carried out using the manufacturer’s instructions as follows. Initial denaturation, 95 °C for 15 min; denaturation, 95 °C for 10 s; annealing, 60 °C for 15 s; elongation, 72 °C for 15 s. The cycle threshold (Ct) values obtained from amplifying *sul1* cDNA were normalized to Ct values of the reference gene *rpoB* using the 2^−ΔΔCt^ method in triplicate. The primers used for real-time PCR are described in Table S3.

### Genome sequencing and read assembly

Genomic DNA (15 μg) that had passed the quality control criteria was sheared into fragments of 15 Kb or larger using a Covariis g-TUBE and purified using 0.45X AMPure XP magnetic beads. Fragment size was measured using a bioanalyzer. Single strand DNA was removed from the end of the DNA strands by incubating the sheared DNA with NAD + , DNA Prep Buffer, DNA Prep Enzyme, and DNA Prep Additive at 37 °C for 15 min. DNA damage was repaired by incubating the DNA with DNA damage repair mix at 37 °C for 30 min. After adding the End Repair Mix, the reaction was allowed to proceed at 20 °C for 10 min and then 65 °C for 30 min. Overhang adapters were added to ends of the end repaired DNA by incubating the adapter, Ligation Mix, Additive, and Enhancer with End Repaired DNA at 20 °C for 60 min and then 65 °C for 10 min. After adding the enzyme clean up kit to the library DNA to which the Overhang adapter had been attached, it reacts at 37 °C for an hour. After purifying the library with 0.45X volume AMPureXP magnetic beads, the amounts and sizes of the DNA recovered were measured using a bioanalyzer. The manufactured library containing 2 to 5 μg DNA was placed in one lane of a BluePippin 0.75% Gel and BP end was set to 13,000 bp and BP start to 9,000 bp. Electrophoresis was performed to collect libraries containing DNA over 9–13 Kb and 15 Kb. The recovered library was refined using 0.5X AMPureXP magnetic beads and the size and density of the library DNA were measured using a bioanalyzer. To improve the overall quality of the reads, the subread data produced by sequencing were converted to HiFi reads using Pacbio CCS version 6.2.0 (https://ccs.how/). De novo assembly was conducted using Flye version 2.8.3 (https://github.com/fenderglass/Flye) assembly tool.

### Bioinformatics analysis

Plasmid replicon-associated genes were analyzed using the PlasmidFinder (https://cge.cbs.dtu.dk/services/PlasmidFinder/) enterobacterial databases with a minimum sequence identity of 95% and minimal gene length coverage of 60% [11, 12]. Isfinder (https://isfinder.biotoul.fr/) was used to identify mobile elements in the plasmid [13]. BLASTN (https://blast.ncbi.nlm.nih.gov/Blast.cgi?PROGRAM=blastn&PAGE_TYPE=BlastSearch&LINK_LOC=blasthome) was used to identify the species with the highest similarity in the NCBI *Salmonella* (taxid: 590) or *Salmonella* Gallinarum (taxid: 594) databases. CLUSTALW (https://www.genome.jp/tools-bin/clustalw) was used for multiple sequence alignments. Artemis (18.2.0) (http://sanger-pathogens.github.io/Artemis/Artemis/) and Snapgene viewer 6.0 (https://www.snapgene.com/snapgene-viewer) were used to visualize multiple alignment results and gene features. GraphPad Prism 9 (https://www.graphpad.com/scientific-software/prism/) was used to present the data.

## Results and discussion

**Fig. 1:**
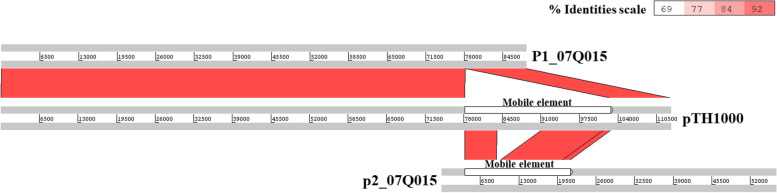
Alignment of pTH1000 and two plasmids p1 (Genbank: CP077761.1) and p2 (Genbank: CP077762.1) in SG_07Q015*.* The nucleotide sequence of the P1 plasmid shares 78% query coverage with that of pTH1000. The sequences share 99% identity. The uncovered region was detected as a Tn3-like mobile element (white color), which was also present in the P2 plasmid with some variation

**Fig. 2:**
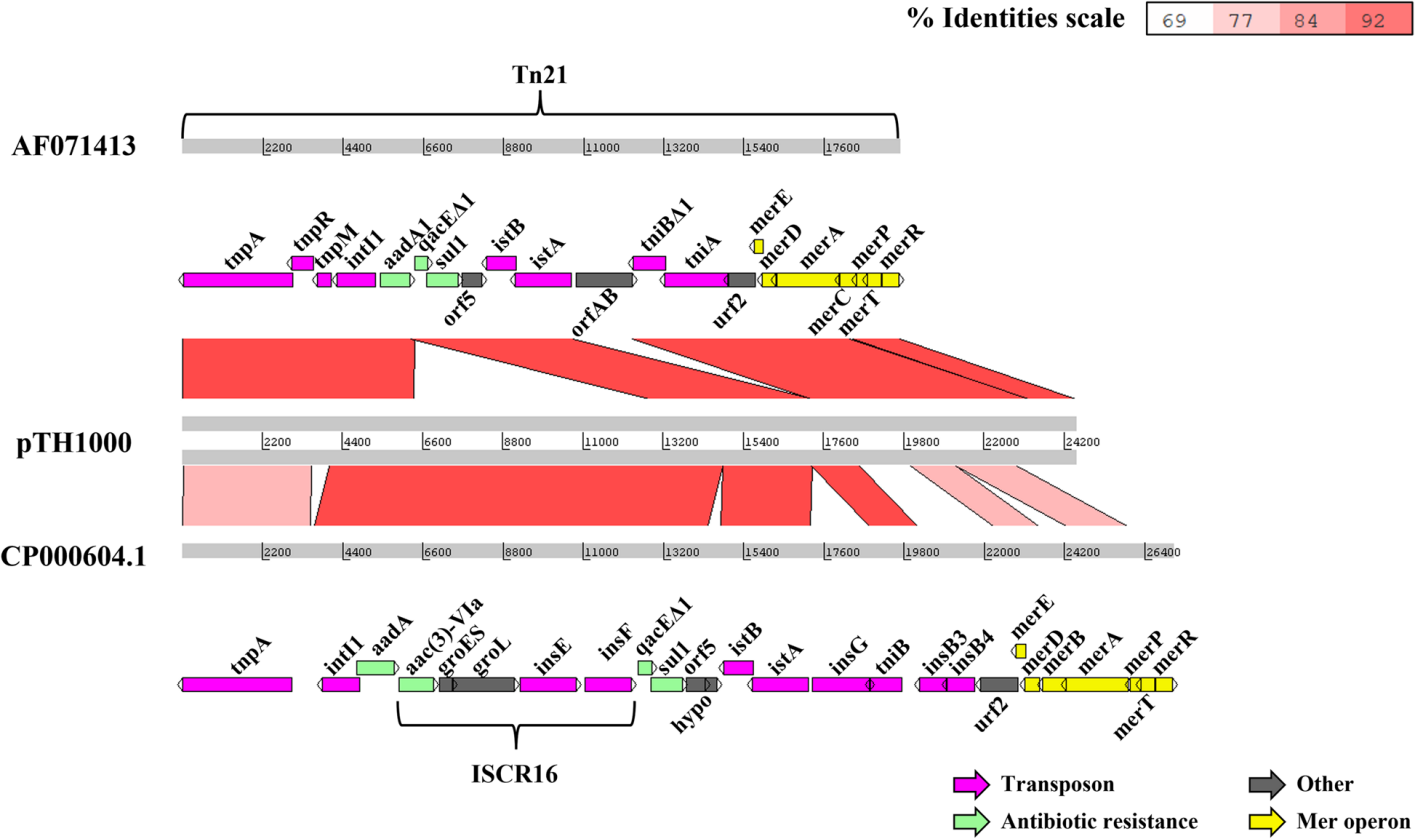
Multiple alignment of the mobile element encoded by plasmid pTH1000 and the Tn21 (Genbank: AF071413) and ISCR16 reference (Genbank: CP000604.1)

*S*. Gallinarum strain SG4021 described in this study was isolated from the liver of a chicken presenting with fowl typhoid in a South Korean broiler farm and identified by the modified rapid slide agglutination test [11]. The genome sequence of SG4021 was determined and deposited in the NCBI GenBank database under accession number CP100648-CP100649. Sequence analysis revealed that SG4021 carries a mega plasmid (112,953 bp, pTH1000). Further analysis demonstrated that its replicon belongs to the incFII(S) family (Figure S2). Genbank contains genome sequences of 63 strains of serovar Gallinarum under taxonomy identifier 594 (taxid 594). In this study, we compared the sequence of plasmid pTH1000 with similar plasmids present in taxid 594. Interestingly, sequence alignment demonstrated similarity with two plasmids (P1 and P2) from strain SG_07Q015, which was also isolated in South Korea and deposited in the Korea Veterinary Culture Collection (Kimchun, Republic of Korea) [9], in addition to other strains (Fig. 1). The sequence of plasmids P1 and P2 is listed in the DDBJ/EMBL/GenBank databases under accession numbers CP077761 and CP077762, respectively. With the exception of a 24.5 kb DNA region containing antibiotic resistant genes, plasmids P1 and pTH1000 shared high query coverage of 78% with a 99.9% identity percentage [9]. Plasmids P1 and pTH1000 carried the incFII(S) replicon, while plasmid P2 carried IncX1 replicon [12, 14]. We found that *Salmonella enterica* subsp. *enterica* serova Schwarzengrun strain CVM N17S1304 isolate 17GA11GT12-S2 (GenBank number CP082637.1) also carried the 24.5 kb sequence present in pTH1000 (Figure S3). Analysis using Isfinder revealed that this 24.5 kb region contained a Tn3-like transposable element [UniProtKB—A0A089VEU8 (A0A089VEU8_SALTI)] [1, 13, 15]. The Tn3-like transposable element is closely related to the Tn21 transposon. In pTH1000, identical 38 bp inverted repeats (IRs) to that of Tn21 were identified at each end of the 24.5 kb region (Figure S4) [16]. Analysis of the Tn3-like transposable element in pTH1000 identified the transposase (*tnpA*), together with transposon modulator (*tnpM*) and resolvase (*tnpR*) clustered at one end, closely resembling the Tn21 reference (AF071413). Insertion sequence IS*1326*, the *tni* module (transposition of the integron), and mercury resistance operon (*mer*) were also identified. The Tn3-like region carried the ISCR16 element (reference CP000604.1) inserted at the In2 variable region but not insertion sequence IS1353 (containing *orfA* and *orfB*), as is observed in different Tn3 subfamilies (Fig. 2) [1]. The *mer* operon (*mer*) was composed of a metal-responsive regulator (*merR*), a coregulator (*merD*), a transport system for delivering mercuric ions across bacterial cytoplasmic membranes (*merTP* and *merE*), and a cytoplasmic mercuric reductase (*merA*) that reduces toxic Hg^2+^ ions [17].

**Fig. 3:**
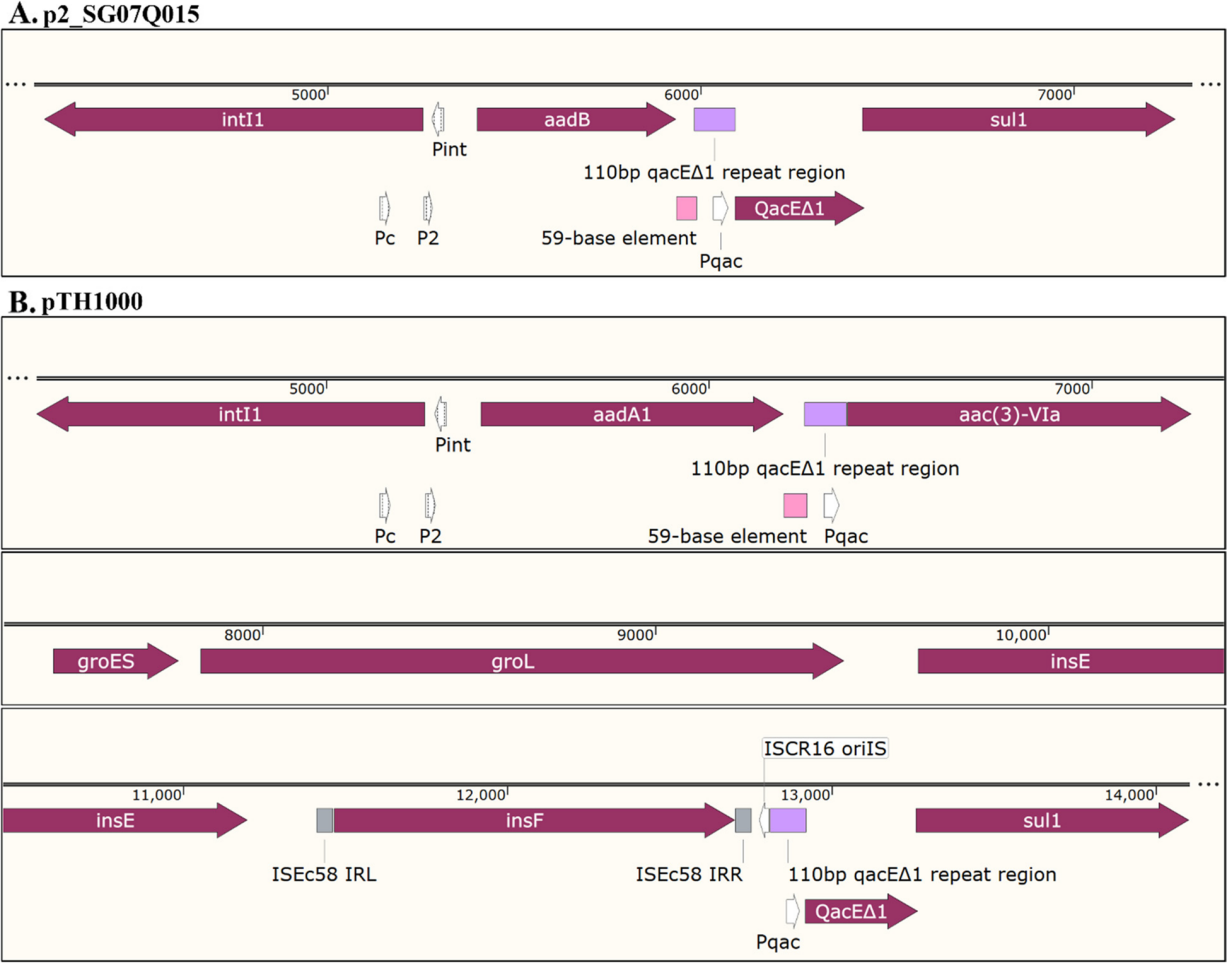
The structure of In2 in plasmid p2 (**A**) in strain SG_07Q015 (Genbank: CP077762.1) and plasmid pTH1000 (**B**). Putative promoters such as Pc and P2 and others are indicated

The same 24.5 kb sequence was found in the P2 plasmid with some notable variations (Fig. 1). The variable region of plasmid P2 in SG_07Q015 contained In2 [18], which encoded an *aadB* gene cassette consisting of an aminoglycoside adenylyltransferase conferring resistance to kanamycin, gentamicin, and tobramycin [19], a variant of the 59-base element (a recombination hot site), followed by *qacEΔ1* conferring resistance to quaternary ammonium compound disinfectants and the *sul1* gene conferring sulfonamide resistance (Fig. 3 and Figure S5) [1, 20]. In pTH1000, an intact ISCR16 (GenBank: CP000604 [21]) sequence is present downstream of the *aadA1* cassette, which contains a small section of *qacEΔ1* (110 bp), *aac(3)-*VIa, *groES/groEL*, *insE*, and *insF*, followed by another *qacEΔ1* and *sul1* sequestered within In2 (Fig. 3). The *aadA1* gene product confers resistance to the two chemically dissimilar drugs streptomycin and spectinomycin by catalyzing the magnesium-dependent transfer of adenosine monophosphate from ATP to the ring structure present in both drugs [22, 23]. The *aac(3)-*VIa gene encodes a 3-N-acetyltransferase that modifies gentamycin and other aminoglycosides [24] (PMID: 16331988). *sul1* confers sulfonamide resistance [2]. No homology was detected between the *aadA1* and the *aadB* coding regions, although these gene cassettes are found at the same location downstream of *intI1*.

**Fig. 4:**
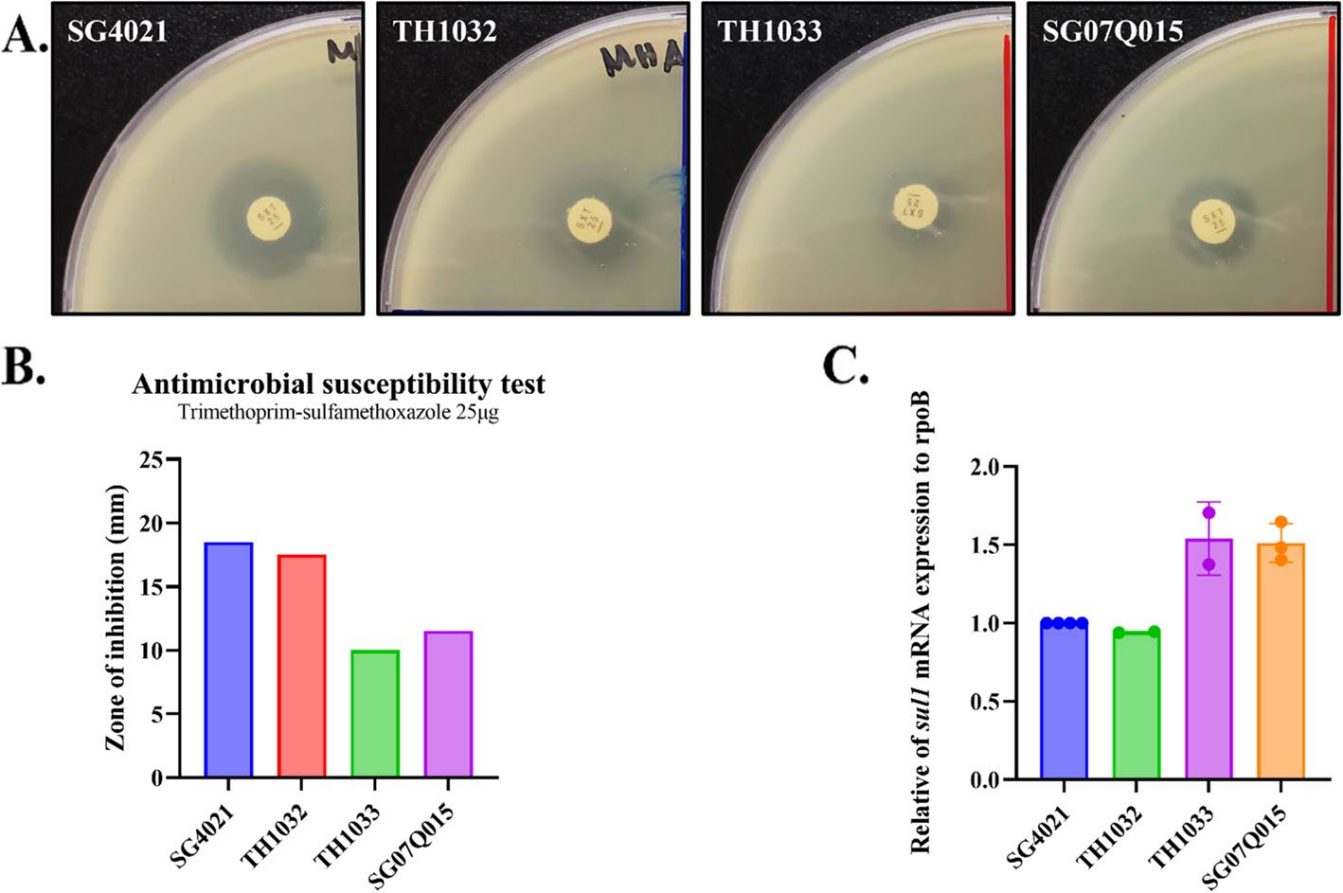
Antibiotic susceptibility of *Salmonella* enterica serovar Gallinarum carrying mutations in the region of the antibiotic cassette of pTH1000. **A** Representative plate images. **B** Quantification of the zones of inhibition. **C** Relative *sul1* mRNA expression determined by qPCR

Several studies have investigated promoter(s) that drive expression of antibiotic resistance genes. Transcription of the *aadA1* cassette was shown to initiate from two promoters located upstream of *intI1*: Pc (also known as Pant or P1) and P2, which contains three guanine residues in the promoter spacer region (Figure S5) [1]. Expression of resistance cassettes from these promoters is a common feature of In2 in many bacteria, including those found in serovar Gallinarum. Further, transcription from these promoters is constitutive [25]. Fifty-nine-base elements are also present immediately downstream of *aadA1* (4, 8, 10) and are imperfect IRs, which have the potential to form stem-loop structures once transcribed and may cause premature transcription termination [25]. *aac(3)-*VIa is frequently present downstream of the 59-base element in the contexts of integrons. Expression of *aac(3)-*VIa downstream of the 59-base element was suggested to be driven by its own promoter [24]. The *sul1* gene is often carried by many resistance plasmids in *Enterobacteriaceae* and is often found in transposons including Tn2Z [26] or Tn2603 [27], which frequently also encode *aadA1*. Resistance plasmid-borne *sul1* is thought to be transcribed from the promoter upstream of *aadA* [28, 29]. In agreement, mutations inactivating the promoter upstream of *aadA* led to loss of downstream *sulI* expression [30], suggesting that Pc/P2 promoters in In2 drive expression of all downstream genes. By contrast, other studies have used nuclease protection assays to demonstrate that expression of *sul1* is driven by its own promoter, *sulZ* [31, 32], although the activity is weak [33]. In this study, we compared strain SG4021 carrying pTH1000, in which a ~ 5 kb ISCR16 is inserted between *aadA1* and *sul1* in the Tn3-like transposable element, and strain SG_07Q015, which does not harbor an insertion sequence between *aadB* and *sul1* in the P2 plasmid. Antibiotic disk diffusion assays showed that strain SG4021 was sensitive to the sulfonamide, while SG_07Q015 was resistant (Fig. 4). To identify the promoter element responsible for expression of *sul1*, two other variant plasmids were constructed from the parental pTH1000 using gene replacement technique [10]. In plasmid pTH1032, both Pc and P2 promoters were removed. In plasmid pTH1033, the sequence between these two promoters and *sul1* was replaced with a *cat* gene lacking a functional promoter. This recombinant sequence resembles that in plasmid P2 from SG_07Q015 in which the Pc/P2 promoter would express both *sul1* and *aadB*. Antibiotic disk diffusion assays revealed that SG4021 carrying pTH1032 was sensitive to sulfonamide, while the strain carrying pTH1033 was resistant (Fig. 4). Therefore, it is unlikely that *sul1* is expressed from its own promoter. Rather, *sul1* is expressed from the Pc/P2 promoters within *intI1*. Next, *sul1* expression in the SG4021 carrying the different constructs was assessed by qPCR. *sul1* expression in SG_07Q015 and SG4021 carrying pTH1033 was higher than those carrying pTH1000 or pTH1032 (Fig. 4).

Within the In2 integron, *sul1* is located downstream of *aadA*, adjacent to the 59-base element and *qacEΔ1* (110 bp) as in P2 plasmid from SG_07Q015. This strain is resistant to sulfonamides, presumably due to expression of *sul1* from Pc and P2. In pTH1000, the insertion element IS*CR16* is present downstream of *aadA1* gene cassette, which places the *sull1* gene ~ 5 kb bp further downstream. Presumably, the IS*CR16* contains a motif for transcription termination for that initiated from Pc/P2. These data warrant caution in predicting antibiotic resistance phenotypes based solely on DNA sequence analysis.

## Conclusion

Tn*3* family integrons and integrated cassette-associated resistance genes are well characterized in Gram-negative pathogens [34]. We compared two isolates of serovar Gallinarum from Korean chicken farms and discovered that strain SG_07Q015 is resistant to kanamycin, gentamicin, tobramycin (*aadB*), and sulfonamide (*sul1*), while strain SG4021 is resistant to streptomycin, spectinomycin (*aadA1*), and gentamycin (*aac(3)-*VIa), but not to sulfonamide [35] (Figure S6). In the poultry industry, gentamicin was recommended for the prevention of early mortality associated with *Salmonella spp.* [36], although two *S*. Gallinarum strains isolated in Korea are already resistant. Sulfonamides are one of the first classes of antimicrobial compounds synthesized early in the twentieth century and have a broad spectrum of bacteriostatic activity against both Gram-positive and negative bacteria and many protozoan organisms. Sulfonamides were used widely in food-producing animals because of their relatively low cost and ease of administration. However, the US Food and Drug Administration withdrew use of sulfonamides in 1990 due to carcinogenic and thyrotoxic activities [37].

According to a survey conducted in Korea reported by [38], almost half of all *S.* Gallinarum isolates collected from poultry farms with fowl typhoid outbreaks exhibited resistance to ampicillin and tetracycline, which reflects the wide use of these antimicrobials in the Korean broiler industry. Interestingly, however, the same survey reported that no Salmonella isolates exhibited resistance to ciprofloxacin, trimethoprim/sulfamethoxazole (Bactrim), or ceftriaxone. Similar results were obtained in a survey of *S.* Gallinarum isolates between 2013 and 2018 [6]. The absence of resistance to these antimicrobials during this period may be because the Korea veterinary antimicrobial resistance system (KVARS) banned the use of sulfonamides and other antimicrobials in formula feed preparations in 2005, which would have resulted in the loss of selective pressure for the maintenance of the integrity of the *sul1* in the course of eventual gene deletion.

## Supplementary Information

**Additional file 1:** **Table S1. **Oligonucleotides usedto introduce mutations upstream of sul1*.*

**Additional file 2:** **Table S2. **Oligonucleotides usedto confirm gene knockout strains*.*

**Additional file 3:** **Table S3.** Oligonucleotides used for real-time PCR*.*

**Additional file 4:** **Figure S1.** Genetic structure ofthe Tn3-like element in Salmonella strains used in this study. Dotted red boxes show promoters that maydrive expression of antibiotic gene cassette. Dotted blue boxes show antibioticgene cassettes. **Figure S2.**Replicon prediction using PlasmidFinder [12,14]. Plasmid replicon-associated DNA sequences were analyzed withPlasmidFinder using the enterobacterial database with a minimum sequenceidentity of 95% and minimal gene length coverage of 60%. Identical nucleotidesare shown with dots, while variant nucleotides are indicated by their singleletter codes. **Figure S3.** Structural alignment of the mobile element inpTH1000, pN17S1304-1 from Salmonella enterica subsp. enterica serovarSchwarzengrund strain CVM N17S1304 isolate 17GA11GT12-S2 (GenBank: CP082637.1),and p2 (Genbank: CP077762.1) from strain SG_07Q015. **Figure S4.** Alignmentof the 38 bp inverted repeat sequences of transposon Tn21 and that present inplasmid pTH1000. Identicalnucleotides are indicated with dots*. ***Figure S5. **Alignmentof DNA sequences adjacent to the intI1 gene in pTH1000 and P2 from strainSG_07Q015 (Genbank: CP077762.1). The promoter element Pc (indicated by a red box) carries twonucleotide variants. Three G residues are inserted in P2 relative to pTH1000 (indicatedby a green box). The 59-base element from P2 carries a 6 nucleotide 5’-GTCTAA-3’ deletion (indicated by a blue box). **Figure S6.** Diskdiffusion antibiotic susceptibility test. (A) Quantification of the zone ofinhibition is indicated for streptomycin (A) and gentamycin (B).

## Data Availability

Not applicable.
