# Supplementary material for: Analysis of antibiotic resistance gene cassettes in a newly identified Salmonella enterica serovar Gallinarum strain in Korea
Source: Mob DNA. 2023 Apr 24;14:4. doi: 10.1186/s13100-023-00292-8 (PMC10124037; doi:10.1186/s13100-023-00292-8)
Supplement: Supplementary file 1 — Additional file 1: Table S1. Oligonucleotides usedto introduce mutations upstream of sul1. [file 13100_2023_292_MOESM1_ESM.docx]

**Table S1.** Oligonucleotides used to introduce mutations upstream of sul1

| **Strain** | **Primer** | **Sequence** |
| --- | --- | --- |
| TH1033 | MultiAntibiotic_res_CM.FOR | 5’-**GCA ACG ATG TTA CGC AGC AGG GCA GTC GCC CTA AAA CAA AGT TAA ACA TC**A TGG AGA AAA AAA TCA CTG GAT ATA CCA CCG-3’ |
|  | Insertion_sul1_pKD3.REV | 5’-**CTT GAC CTG ATA GTT TGG CTG TGA GCA ATT ATG TGC TTA GTG CAT CTA AC**G TGT AGG CTG GAG CTG CTT C-3’ |
| TH1032 | pSulI_pKD3.FOR | 5’-**ATG CTC GCC TTC CAG AAA ACC GAG GAT GCG AAC CAC TTC ATC CGG GGT CA**G TGT AGG CTG GAG CTG CTT C-3’ |
|  | pSulI_pKD3.REV | 5’-**AGG GCG ACT GCC CTG CTG CGT AAC ATC GTT GCT GCT CCA TAA CAT CAA AC**G GTC CAT ATG AAT ATC CTC CTT AGT TCC TAT TCC-3’ |

**a. Homologous sequences for recombination are shown as bold and underlined.**
