# Supplementary material for: Analysis of antibiotic resistance gene cassettes in a newly identified Salmonella enterica serovar Gallinarum strain in Korea
Source: Mob DNA. 2023 Apr 24;14:4. doi: 10.1186/s13100-023-00292-8 (PMC10124037; doi:10.1186/s13100-023-00292-8)
Supplement: Supplementary file 2 — Additional file 2: Table S2. Oligonucleotides usedto confirm gene knockout strains. [file 13100_2023_292_MOESM2_ESM.docx]

**Table S2.** Oligonucleotides used to confirm gene knockout strains

| **Strain** | **Primer** | **Sequence** |
| --- | --- | --- |
| TH1033 | MultiAntibiotic_res.FOR | 5’-GCA GTC GCC CTA AAA CAA AGT TAA ACA TC-3’ |
|  | MultiAntibiotic_res.REV | 5’-GCG GCC GGA AGG TGA ATG-3’ |
| TH1032 | pSulI.FOR | 5’-AGA AGC TGG GCG AAC AAA CG-3’ |
|  | pSulI.REV | 5’-CGC TTC CCT CAT GAT GTT TAA CTT TGT TTT-3’ |
