# Supplementary material for: Analysis of antibiotic resistance gene cassettes in a newly identified Salmonella enterica serovar Gallinarum strain in Korea
Source: Mob DNA. 2023 Apr 24;14:4. doi: 10.1186/s13100-023-00292-8 (PMC10124037; doi:10.1186/s13100-023-00292-8)
Supplement: Supplementary file 3 — Additional file 3: Table S3. Oligonucleotides used for real-time PCR. [file 13100_2023_292_MOESM3_ESM.docx]

**Table S3.** Oligonucleotides used for real-time PCR

| **Gene** | **Primer** | **Sequence** |
| --- | --- | --- |
| rpoB | SG_rpoB_FW | 5’-GCG TCT CAA GGA AGC CAT ATT C-3’ |
|  | SG_rpoB_RV | 5’-GTC GCG TAT GTC CTA TCG AAA C-3’ |
| sul1 | qPCR_SGsul1.FOR | 5’-CGC ACC GGA AAC ATC GCT GCA C-3’ |
|  | qPCR_SGsul1.REV | 5’-TGA AGT TCC GCC GCA AGG CTC G-3’ |
