# Supplementary material for: Analysis of antibiotic resistance gene cassettes in a newly identified Salmonella enterica serovar Gallinarum strain in Korea
Source: Mob DNA. 2023 Apr 24;14:4. doi: 10.1186/s13100-023-00292-8 (PMC10124037; doi:10.1186/s13100-023-00292-8)
Supplement: Supplementary file 4 — Additional file 4: Figure S1. Genetic structure ofthe Tn3-like element in Salmonella strains used in this study. Dotted red boxes show promoters that maydrive expression of antibiotic gene cassette. Dotted blue boxes show antibioticgene cassettes. Figure S2.Replicon prediction using PlasmidFinder [1214]. Plasmid replicon-associated DNA sequences were analyzed withPlasmidFinder using the enterobacterial database with a minimum sequenceidentity of 95% and minimal gene length coverage of 60%. Identical nucleotidesare shown with dots, while variant nucleotides are indicated by their singleletter codes. Figure S3. Structural alignment of the mobile element inpTH1000, pN17S1304-1 from Salmonella enterica subsp. enterica serovarSchwarzengrund strain CVM N17S1304 isolate 17GA11GT12-S2 (GenBank: CP082637.1),and p2 (Genbank: CP077762.1) from strain SG_07Q015. Figure S4. Alignmentof the 38 bp inverted repeat sequences of transposon Tn21 and that present inplasmid pTH1000. Identicalnucleotides are indicated with dots. Figure S5. Alignmentof DNA sequences adjacent to the intI1 gene in pTH1000 and P2 from strainSG_07Q015 (Genbank: CP077762.1). The promoter element Pc (indicated by a red box) carries twonucleotide variants. Three G residues are inserted in P2 relative to pTH1000 (indicatedby a green box). The 59-base element from P2 carries a 6 nucleotide 5’-GTCTAA-3’ deletion (indicated by a blue box). Figure S6. Diskdiffusion antibiotic susceptibility test. (A) Quantification of the zone ofinhibition is indicated for streptomycin (A) and gentamycin (B). [file 13100_2023_292_MOESM4_ESM.docx]

*
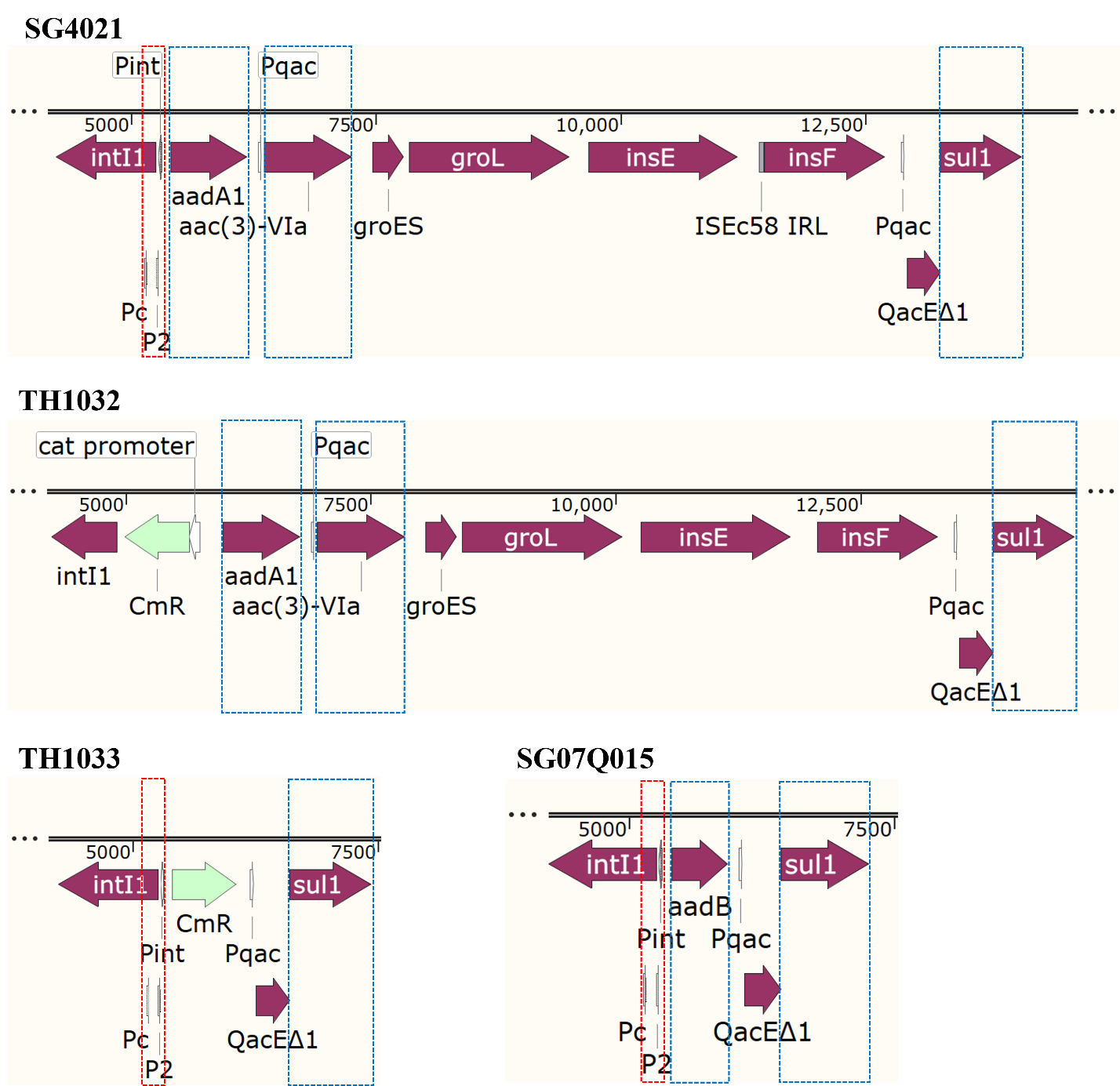
*

**Figure S1.** **Genetic structure of the Tn3-like element in Salmonella strains used in this study**. Dotted red boxes show promoters that may drive expression of antibiotic gene cassette. Dotted blue boxes show antibiotic gene cassettes.

**
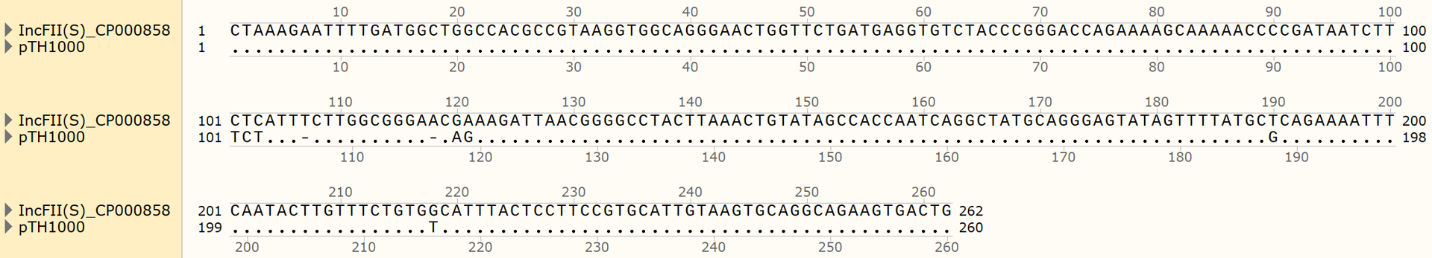
**

**Figure S2. Replicon prediction using PlasmidFinder** [[12](#_ENREF_12), [14](#_ENREF_14)]. Plasmid replicon-associated DNA sequences were analyzed with PlasmidFinder using the enterobacterial database with a minimum sequence identity of 95% and minimal gene length coverage of 60%. Identical nucleotides are shown with dots, while variant nucleotides are indicated by their single letter codes.


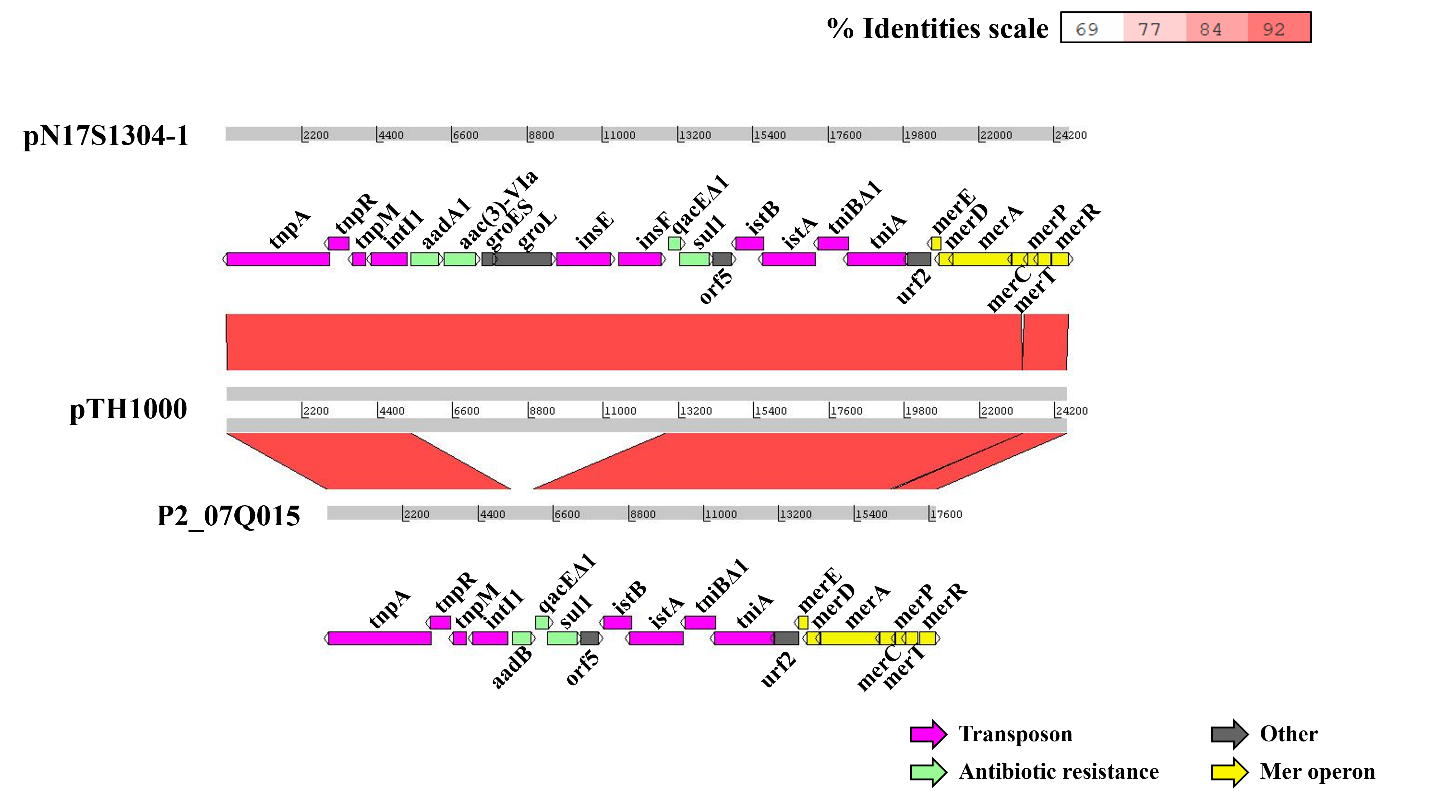


**Figure S3. Structural alignment of the mobile element in pTH1000, pN17S1304-1 from Salmonella enterica subsp. enterica serovar Schwarzengrund strain CVM N17S1304 isolate 17GA11GT12-S2 (GenBank: CP082637.1), and p2 (Genbank: CP077762.1) from strain SG_07Q015.**

**
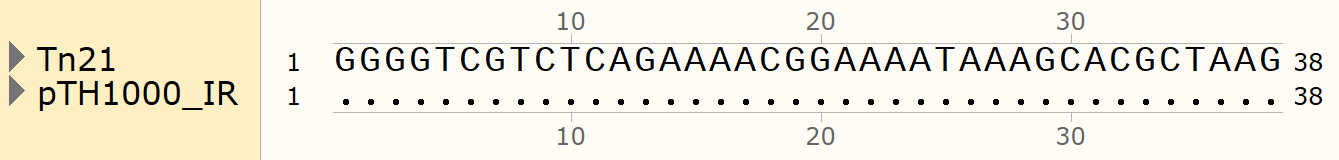
**

**Figure S4**. **Alignment of the 38 bp inverted repeat sequences of transposon Tn21 and that present in plasmid pTH1000**. Identical nucleotides are indicated with dots.

***
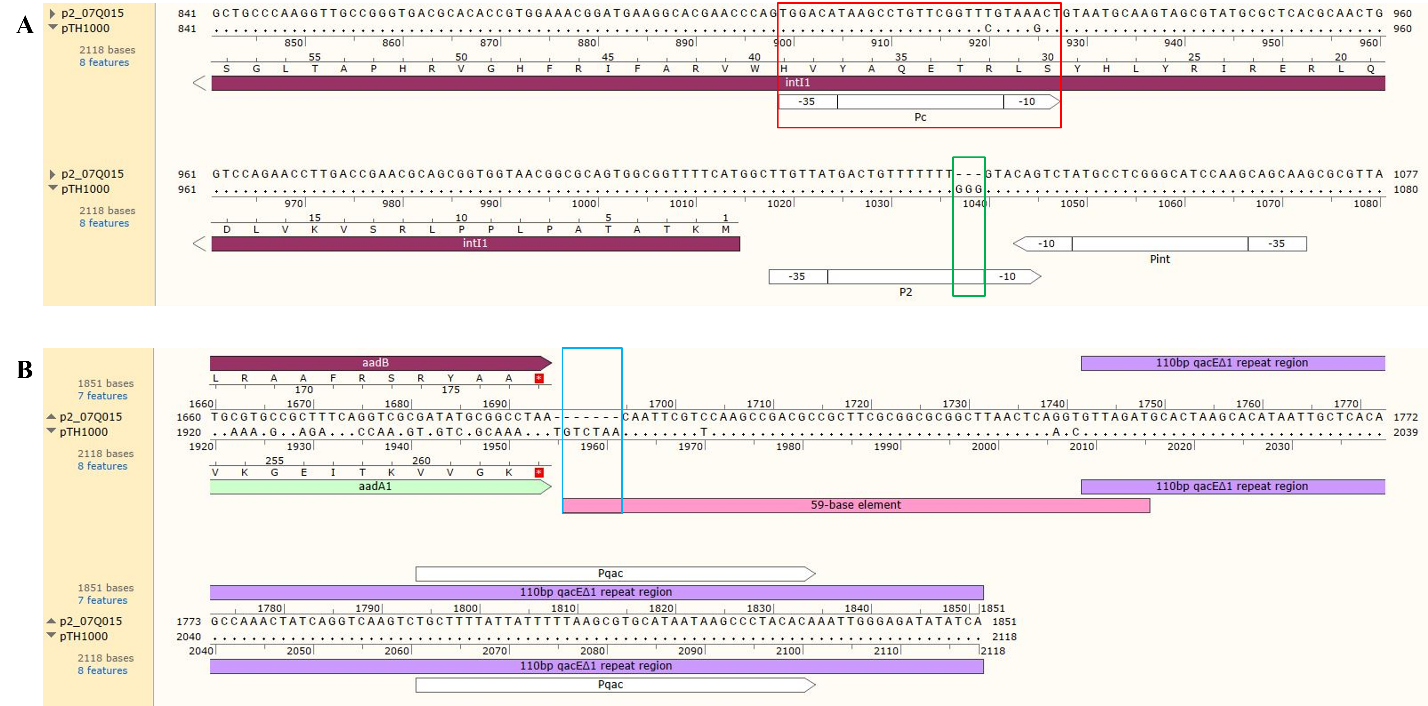
***

**Figure S5.** **Alignment of DNA sequences adjacent to the intI1 gene in pTH1000 and P2 from strain SG_07Q015 (Genbank: CP077762.1).** The promoter element Pc (indicated by a red box) carries two nucleotide variants. Three G residues are inserted in P2 relative to pTH1000 (indicated by a green box). The 59-base element from P2 carries a 6 nucleotide 5’-GTCTAA-3’ deletion (indicated by a blue box).


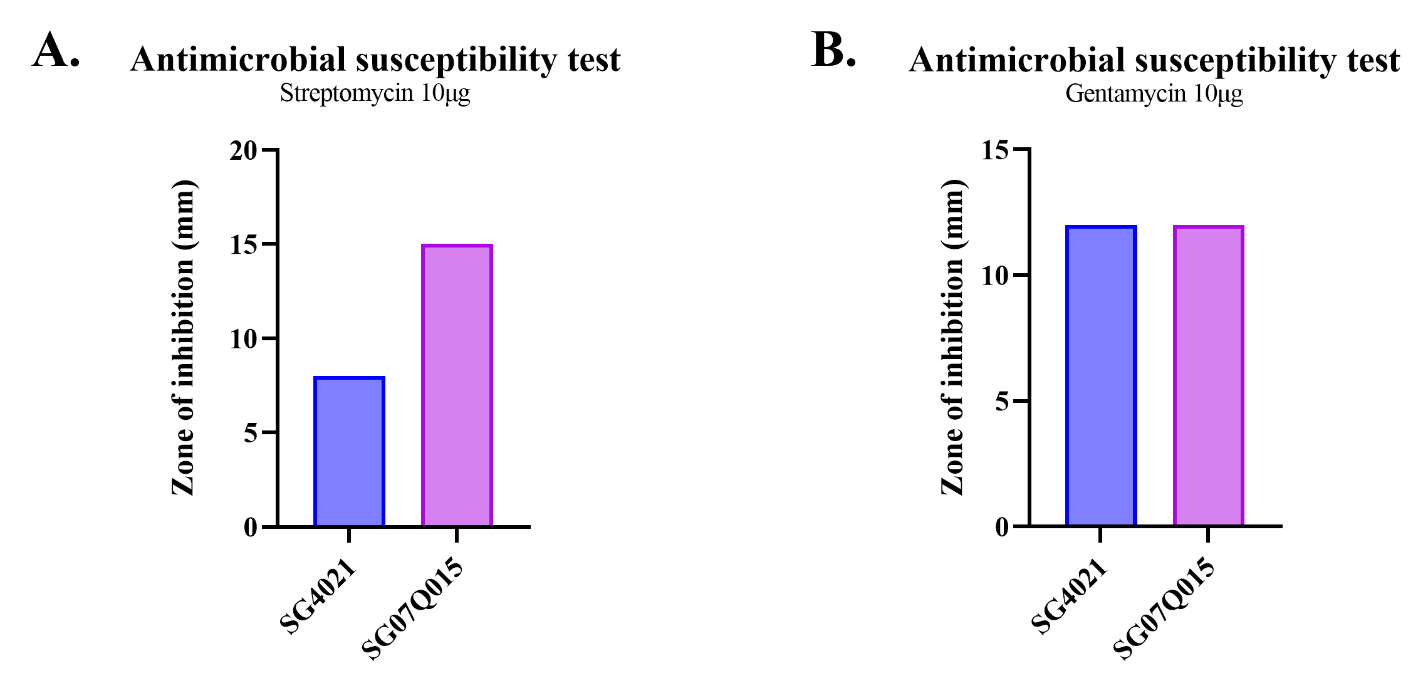


**Figure S6**. **Disk diffusion antibiotic susceptibility test**. (A) Quantification of the zone of inhibition is indicated for streptomycin (A) and gentamycin (B).
